# What shapes template-matching performance in cryogenic electron tomography *in situ*?

**DOI:** 10.1107/S2059798324004303

**Published:** 2024-05-28

**Authors:** Valentin J. Maurer, Marc Siggel, Jan Kosinski

**Affiliations:** a European Molecular Biology Laboratory Hamburg, Notkestrasse 85, 22607 Hamburg, Germany; b Centre for Structural Systems Biology (CSSB), Notkestrasse 85, 22607 Hamburg, Germany; c Structural and Computational Biology Unit, European Molecular Biology Laboratory, Meyerhofstrasse 1, 69117 Heidelberg, Germany; National Centre for Biological Sciences-TIFR, India

**Keywords:** cryo-electron tomography, template matching, computer vision, particle picking

## Abstract

The detection of specific biological macromolecules in cryogenic electron tomography data is frequently approached by applying cross-correlation-based 3D template matching. To reduce computational cost and noise, high binning is used to aggregate voxels before template matching. This remains a prevalent practice in both practical applications and methods development. Here, the relation between template size, shape and angular sampling is systematically evaluated to identify ribosomes in a ground-truth annotated data set. It is shown that at the commonly used binning, a detailed subtomogram average, a sphere and a heart emoji result in near-identical performance. These findings indicate that with current template-matching practices macromolecules can only be detected with high precision if their shape and size are sufficiently different from the background. Using theoretical considerations, the experimental results are rationalized and it is discussed why primarily low-frequency information remains at high binning and that template matching fails to be accurate because similarly shaped and sized macromolecules have similar low-frequency spectra. These challenges are discussed and potential enhancements for future template-matching methodologies are proposed.

## Abstract 2

The relation between template size, shape and angular sampling is systematically evaluated to identify ribosomes in a ground-truth annotated data set. The findings are discussed in a theoretical framework.

## 1. Introduction

Cellular cryogenic electron tomography (cryo-ET) has emerged as a key method to unravel the structural and spatial complexity of the cell. The 3D volume of a cellular region, called a tomogram, is reconstructed from 2D projection images acquired using a transmission electron microscope in many different orientations (Pyle & Zanetti, 2021 ▸; Volkmann, 2010 ▸). Macromolecular complexes can be identified in the tomogram, their spatial arrangement can be analyzed in the native environment, and their structure can potentially be determined to near-atomic resolution by subtomogram averaging (Mahamid *et al.*, 2016 ▸; Pfeffer *et al.*, 2017 ▸; Wilfling *et al.*, 2020 ▸; Lučić *et al.*, 2013 ▸; Xue *et al.*, 2022 ▸; Hoffmann *et al.*, 2022 ▸).

However, the identification of individual macromolecules in tomograms is challenging due to the missing wedge, which results from limitations on the possible tilt angles of the specimen, a low signal-to-noise ratio due to the use of low electron doses during acquisitions, and the crowded cellular environment of the cell. Because of these complications, segmentation and subsequent analysis of tomograms is still a difficult task and a major bottleneck for fully automated high-throughput analysis of large cryo-ET data sets (Lučić *et al.*, 2013 ▸; Pyle & Zanetti, 2021 ▸; Wu *et al.*, 2019 ▸; de Teresa-Trueba *et al.*, 2023 ▸).

A commonly used approach to identify macromolecules within tomograms is so-called template matching, in which a reference density of a macromolecule is used to localize the corresponding candidate positions within the tomogram (Frangakis *et al.*, 2002 ▸; Böhm *et al.*, 2000 ▸). To date, various packages have been developed to perform template matching, such as *PyTom* (Hrabe *et al.*, 2012 ▸), *STOPGAP* (Wan *et al.*, 2020 ▸, 2024 ▸), *EMAN*2 (Tang *et al.*, 2007 ▸), *DYNAMO* (Castaño-Díez *et al.*, 2012 ▸) and *pyTME* (Maurer *et al.*, 2024 ▸). All of these packages use cross-correlation-based scoring metrics to identify macromolecules in tomograms (see Section 3.4). The templates used for template matching range from simple shapes such as spheres, cylinders and rectangles, which have previously been used to detect various cellular structures (Engel *et al.*, 2015 ▸; Cai *et al.*, 2018 ▸; Nickell *et al.*, 2007 ▸; Lebbink *et al.*, 2007 ▸), to detailed maps obtained from experiments or generated from atomic structures (Frangakis *et al.*, 2002 ▸; Beck *et al.*, 2009 ▸; Kühner *et al.*, 2009 ▸). A common use case is the ribosome, which is abundantly found in tomograms and can often be identified by eye due to its size. However, even for a particle as large as the ribosome, template matching has suboptimal precision (Zhang *et al.*, 2023 ▸; de Teresa-Trueba *et al.*, 2023 ▸). For smaller or less abundant macromolecules, the method often fares even worse and requires manual curation. These points raise the question of what the requirements and limitations are for the reliable use of template matching for macromolecules in *in situ* cryo-ET.

Although tomograms are usually collected at ≤2 Å per voxel, they are typically binned 4–8 times to a coarse voxel size in order to improve the computational efficiency and signal-to-noise ratio (de Teresa-Trueba *et al.*, 2023 ▸; Xue *et al.*, 2022 ▸; Engel *et al.*, 2015 ▸; Cai *et al.*, 2018 ▸; Frangakis *et al.*, 2002 ▸; Chaillet *et al.*, 2023 ▸; Wan *et al.*, 2024 ▸; Rice *et al.*, 2023 ▸; Genthe *et al.*, 2023 ▸; Hoffmann *et al.*, 2022 ▸). However, binning removes high-frequency information from the tomogram and makes it difficult to distinguish macromolecules if the differences in the low-frequency components are not sufficiently large (Böhm *et al.*, 2000 ▸). Therefore, template matching under such settings is prone to have low precision, and manual curation or other means of refinement are required to improve the results. The issue of low precision has been hinted at previously, and one suggested solution is template matching in 2D (Lucas *et al.*, 2021 ▸; Rickgauer *et al.*, 2017 ▸). However, to our knowledge there has been no published study that systematically explores how the choice of an exact template, its size and the degree of angular sampling affect *in situ* template-matching results.

Here, we investigate the pitfalls of 3D template matching with the commonly used four-times binned tomograms and rationalize the observed issues. We assess the precision of detecting ribosomes in a previously annotated tomogram by de Teresa-Trueba *et al.* (2023 ▸) using a detailed subtomogram average of a ribosome, a sphere, a heart emoji and a structure of hemagglutinin at different sizes as templates. We find that at this binning the size and approximate shape are the major determinants of precision, and the exact template choice or angular sampling has little impact on the template-matching results. We then rationalize these observations theoretically by inspecting the Fourier transforms of simple geometric shapes and show that when low-frequency components dominate in the tomogram, similarly sized and shaped templates result in nearly identical template-matching precision. Finally, we discuss the implications of these results for the development and benchmarking of template-matching algorithms as well as requirements for data processing. A further aim of our analysis is to guide optimal experimental design in practical applications and the development of new template-matching methods.

## 2. Methods

**Figure 1:**
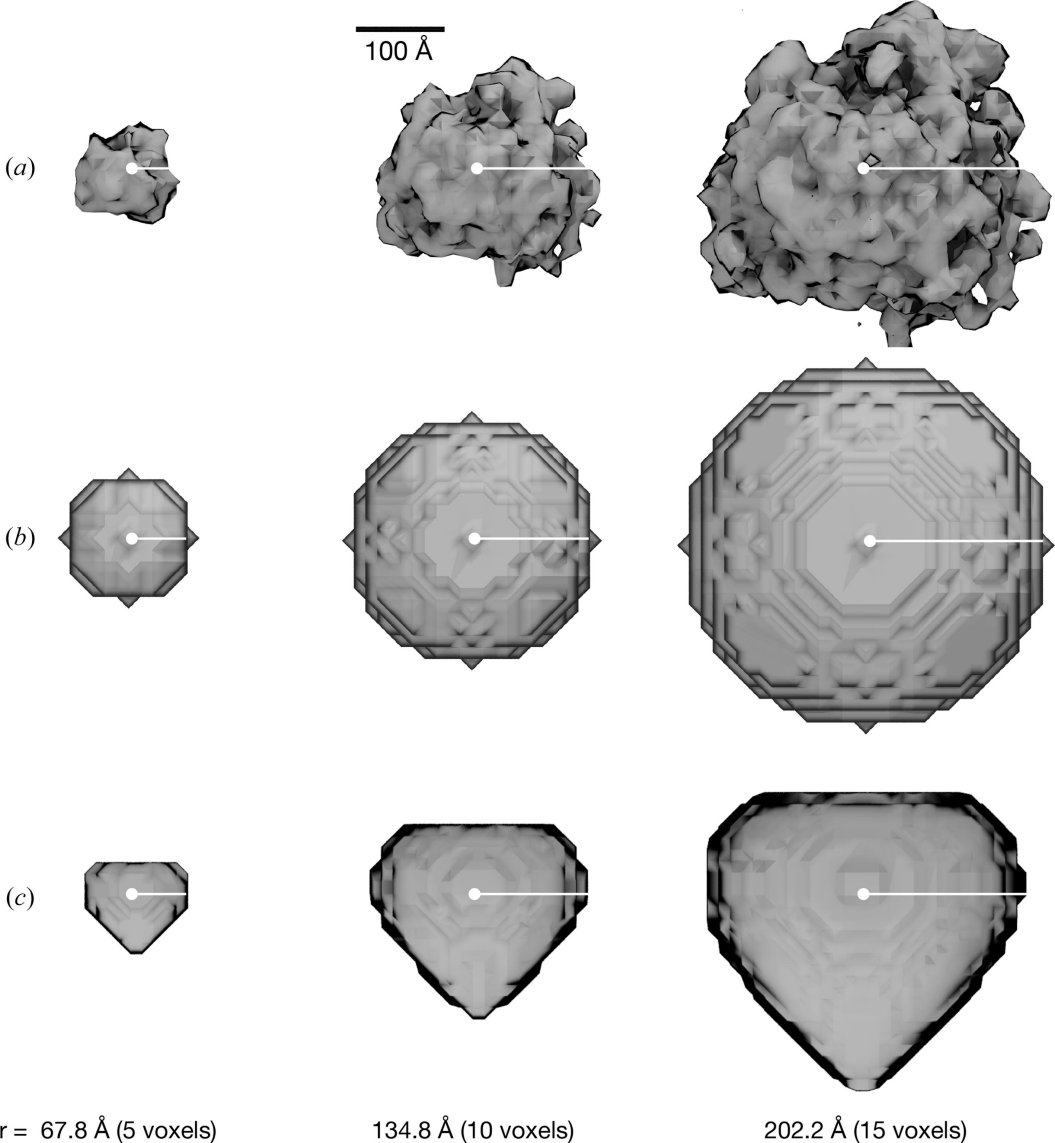
Template classes used to match the ribosome in the previously annotated tomogram from de Teresa-Trueba *et al.* (2023 ▸). Different shapes with different radii sampled at a 13.8 Å voxel size, to match the voxel size of the tomogram, were used as templates for template matching with *PyTom*. Specifically, the map of the 80S ribosome (*a*) (EMDB entry EMD-3228), a sphere (*b*) and a rotationally symmetrized heart emoji (*c*) were used. The used radii range from 1 to 19 voxels in one-voxel increments. Only three representative radii are shown.

All template-matching experiments were performed using *PyTom* (Hrabe *et al.*, 2012 ▸; version 1.0) and cross-validated using *pyTME* (Maurer *et al.*, 2024 ▸; version 0.1.7) on an annotated reconstructed 3D tomogram (EMPIAR-10988, TS_037; Iudin *et al.*, 2022 ▸) reported by de Teresa-Trueba *et al.* (2023 ▸). For this sample tomogram, 1646 ribosomal and 22 fatty-acid synthase (FAS) positions were previously identified using *PyTom* (Hrabe *et al.*, 2012 ▸) and subsequently manually refined by an expert user. Template matching was performed for four different template classes that were provided at varying sizes, as shown in Fig. 1 ▸. Firstly, a previously reported 3D map of the *Saccharomyces cerevisiae* 80S ribosome (EMDB entry EMD-3228; Bharat & Scheres, 2016 ▸) was used as a baseline reference (Fig. 1 ▸ *a*). Secondly, a sphere with varying radius *r* (1 ≤ *r* < 20) and homogenous density (Fig. 1 ▸ *b*) was used. The third template was the heart emoji from the Apple Color Emoji font. The 2D bytemap was converted to a volume with homogenous density by axial symmetrization sampling 360 equidistant angles and was subsequently blurred using a Gaussian filter (*scipy.ndimage.gaussian_filter*, version 1.11.1) with σ = 1 (Fig. 1 ▸ *c*). The heart emoji was scaled from the initial 160 × 160 bytemap to 20 × 20 using linear spline interpolation. As an additional control template with a clearly distinct shape, a structural model of the hemagglutinin trimer was used. The atomic structure was obtained by modeling with *AlphaFold*2-*multimer* (Jumper *et al.*, 2021 ▸) using the A/Hong Kong/1/1968 H3N2 strain. The default parameters were used, with the exception of the number of refinement cycles being increased to 6. The best model was chosen based on the lowest overall predicted aligned error. All templates were placed in the center of a cubic volume with an edge length of 51 voxels and a voxel size of 13.48 Å, corresponding to the voxel size of the used tomogram. The 3D ribosome map and atomic structure were resampled from their respective grids to match the sampling rate of the tomogram (Supplementary Fig. S1*a*). The template contained in each of the four created volumes was assigned a radius of 10, which is approximately equal to the radius of their respective bounding spheres. We simulated different radii of each template by resampling these initial volumes to a sampling rate computed as (10 × 13.48)/radius, *i.e.* a radius of 11 voxels corresponds to 1.1 times the voxel size of the tomogram and results in a 10% larger template. Although principally arbitrary, the factor of 10 was chosen because it is the radius of the bounding sphere of the ribosome at the considered voxel size, *i.e.* at a radius of 10 the ribosome map should exactly represent ribosomes in the tomogram. The sphere template was not obtained by resampling but instead was created directly using the respective radius. The 3D map or structure was not directly sampled on grids of varying voxel sizes to avoid introducing additional detail for higher radii. As a mask, a sphere with a radius two voxels larger than the template radius was used, which is in good concordance with the 337 Å diameter mask used by de Teresa-Trueba *et al.* (2023 ▸). The tilt series underlying the tomogram used here was acquired using tilt angles from −50° to 50°, which corresponds to a 40° wedge angle in the *PyTom* convention (de Teresa-Trueba *et al.*, 2023 ▸; Hrabe *et al.*, 2012 ▸). *PyTom* was instructed to generate a binary wedge mask based on this specification, which is applied to the Fourier transform of the template after each rotation, in order to introduce a missing wedge analogous to the tomogram in the template. *PyTom* samples translations exhaustively (see equation 2) and rotational degrees of freedom uniformly with a given sampling rate using a pre­defined set of rotations. When performing template matching, the template is rotated, translations are sampled exhaustively and high scores are retained. This is repeated for all rotations in the set. *PyTom* outputs a score for each translation and the corresponding sampled rotation of the template used to acquire that score. *pyTME* uses the same overall approach. For the spheres 90° (two angles) was sampled, and for the other templates 25.25° (980 angles), 19.95° (1944 angles) and 11° (15 192 angles) were sampled, which correspond to the angle lists angles_90_2, angles_25.25_980, angles_19.95.25_1944 and angles_11_15192 that are shipped with *PyTom* (Hrabe *et al.*, 2012 ▸). The rotational sampling rate of 11° is in excess of what has typically been used for template matching in previous work (de Teresa-Trueba *et al.*, 2023 ▸; Hrabe *et al.*, 2012 ▸; Pfeffer *et al.*, 2012 ▸). *PyTom* was run assuming a spherical mask, using a bandpass filter with low-frequency and high-frequency cutoffs of 3 and 15, respectively, splitting the tomogram into three parts along each axis and performing no further binning. The results were cross-validated using *pyTME*, which can perform template matching using a similar formulation of the cross-correlation score (Maurer *et al.*, 2024 ▸). *pyTME* was run on the same data, with the difference that no bandpass filter and no wedge mask were applied prior to template matching. 4000 peaks were called on the scores obtained from *PyTom* and *pyTME* using *skimage.feature.peak_local_max* (version 0.21.0), with a minimal allowed Euclidean distance separating peaks of 10 and a 15-voxel exclusion volume around the boundaries of the tomogram. Subsequently, peaks were ordered by their score in decreasing order. The precision and recall were analyzed at decreasing score thresholds up to 4000 top-scoring peaks.

## 3. Results and discussion

### 3.1. Shape and size are the major determinants for template-matching precision

**Figure 2:**
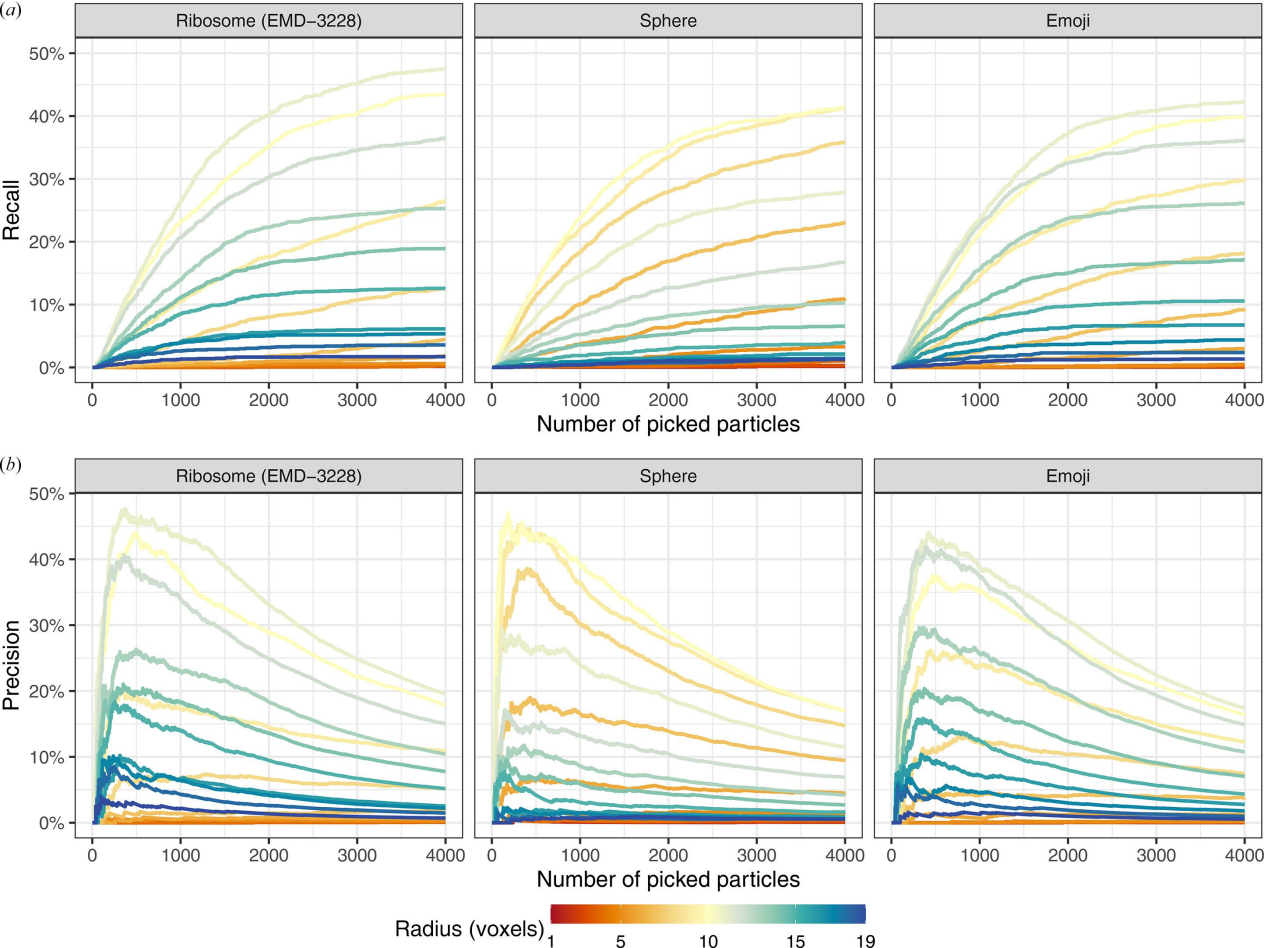
Template-matching performance using three distinct template classes scaled to different radii (see Fig. 1 ▸). Radius scaling was performed by resampling each template to 10 × (radius)^−1^ times the sampling rate of the tomogram, starting from an initial template with an assigned radius of 10 and the same sampling rate as the tomogram (see Section 2). (*a*) Ribosome-picking recall by the number of picked particles. We used linear sum assignment to achieve an optimal one-to-one mapping between ground-truth and picked particles. A particle is considered to be correctly picked if it is within a five-voxel distance of its assigned ground-truth particle. Consequently, all particles without assignment to ground-truth particles were considered to be false positives. All 4000 picked particles were considered in the following figures. (*b*) As in (*a*) but showing precision instead of recall.

To assess how sensitive template matching is *in situ* to the specific shape and size of a template, we used *PyTom* (Hrabe *et al.*, 2012 ▸) to perform template matching with four different templates on a four-times binned tomogram with a voxel size of 13.48 Å reported by de Teresa-Trueba *et al.* (2023 ▸). This is comparable to the voxel sizes typically used in many previously published template-matching studies (de Teresa-Trueba *et al.*, 2023 ▸; Wan *et al.*, 2024 ▸; Xue *et al.*, 2022 ▸; Engel *et al.*, 2015 ▸; Cai *et al.*, 2018 ▸; Frangakis *et al.*, 2002 ▸; Chaillet *et al.*, 2023 ▸; Rice *et al.*, 2023 ▸; Hoffmann *et al.*, 2022 ▸). We also used *pyTME* (Maurer *et al.*, 2024 ▸) to independently cross-validate these results. The tomogram contained 1646 ribosomes and 22 FAS particles, which were annotated by the authors using template matching and manual curation. We consider their annotation as a robust ground truth. As per de Teresa-Trueba *et al.* (2023 ▸), we used an 80S ribosome (Bharat & Scheres, 2016 ▸) map as the initial template and scaled its radius to see how size affects template-matching performance (Fig. 1 ▸ *a*). We also tested spheres and an irregularly shaped heart emoji at various radii (Figs. 1 ▸ *b* and 1 ▸ *c*) to check whether the exact properties of the template are relevant at this binning to achieve high precision in template matching and also compared a variety of angular sampling rates. Spheres have already successfully been used in practice to identify RuBisCO (Engel *et al.*, 2015 ▸), and other basic shapes such as cylinders for nucleosomes (Cai *et al.*, 2018 ▸) or the proteasome (Nickell *et al.*, 2007 ▸) and rectangles for membranes (Lebbink *et al.*, 2007 ▸). However, a side-by-side comparison has been lacking to date. Therefore, we compared the template-matching results for the different templates and scaled radii and angular samplings based on precision [precision = TP/(TP + FP)] and recall [recall = TP/(TP + FN)], where TP, FP and FN correspond to the number of true positives, false positives and false negatives, respectively (Fig. 2 ▸). The picked particles were chosen from a sorted list of all scores in descending order.

Firstly, we compared the recall across the different templates and with different radii calculated with respect to the number of picked particles (Fig. 2 ▸ *a*). Overall, the performance of up to 4000 top-scoring picks across the templates was comparable, with a maximal recall of around 40–50%. The recall was optimal across templates for radii close to 10 and decreased for smaller or larger radii. Since ribosomes in the tomogram have a radius of 10, *i.e.* their bounding sphere has an approximate radius of 10, these results indicated that all templates are capable of matching ribosomes if scaled to the correct radii. Hence, at the voxel size of 13.48 Å used here, a realistic *S. cerevisiae* ribosome map (EMDB entry EMD-3228; Bharat & Scheres, 2016 ▸) performs no better than a sphere or an emoji of similar size on the same data set. Furthermore, given the shape of the curves, it appears unlikely that the remaining ribosomes could be recovered at reasonable precision. Given that no template recapitulated the ground-truth particle set beyond a recall of 50%, it becomes questionable whether using improved experimental or predicted structures as templates will be sufficient to identify small proteins or low-abundance proteins in cryo-ET data at this binning. This is further substantiated by ribosomes already requiring manual curation (de Teresa-Trueba *et al.*, 2023 ▸). The remaining high-scoring picks are likely to correspond to other particles or features of similar shape and size.

Similarly, the precision for the different templates peaked at around ∼750 picked particles independently of the template choice but not the template radius (Fig. 2 ▸ *b*). Picking more than 750 particles improved the overall recall but led to a disproportionate identification of false positives, thus reducing the overall precision. We observed this behavior consistently for all templates, and there was little differentiation between the correct ribosome template and the sphere or emoji template. The precision values observed here are in line with the 19% reported by de Teresa-Trueba *et al.* (2023 ▸) for all ten defocus tomograms. The observed curve shapes could be explained by the existence of distinct ribosome populations that differ in their ability to be identified by template matching. While optimal results are achieved using templates that recapitulate the size of the ribosome, a subset of annotated ribosomes appears to exist that can be identified with incorrect radii.

To further confirm this finding, we also ran control experiments using an Influenza A virus hemagglutinin (HA) template, which has a markedly different shape to a ribosome (Supplementary Fig. S1*a*). HA is a trimer with a total molecular weight of 180 kDa that has an approximately cylindrical shape with a length of ∼17 nm and a width of ∼6 nm. We scaled the radius analogous to the previous structures and calculated the precision with respect to the ground-truth data. The precision was near 0% for sizes up to 10 voxels, and only for larger radii did the precision increase as the structure further approaches the shape and size of the ribosome (Supplementary Fig. S1*b*). This further underscores the observation that at this level of binning, template matching is less dependent on the structure and overall focuses on shape and size.

**Figure 3:**
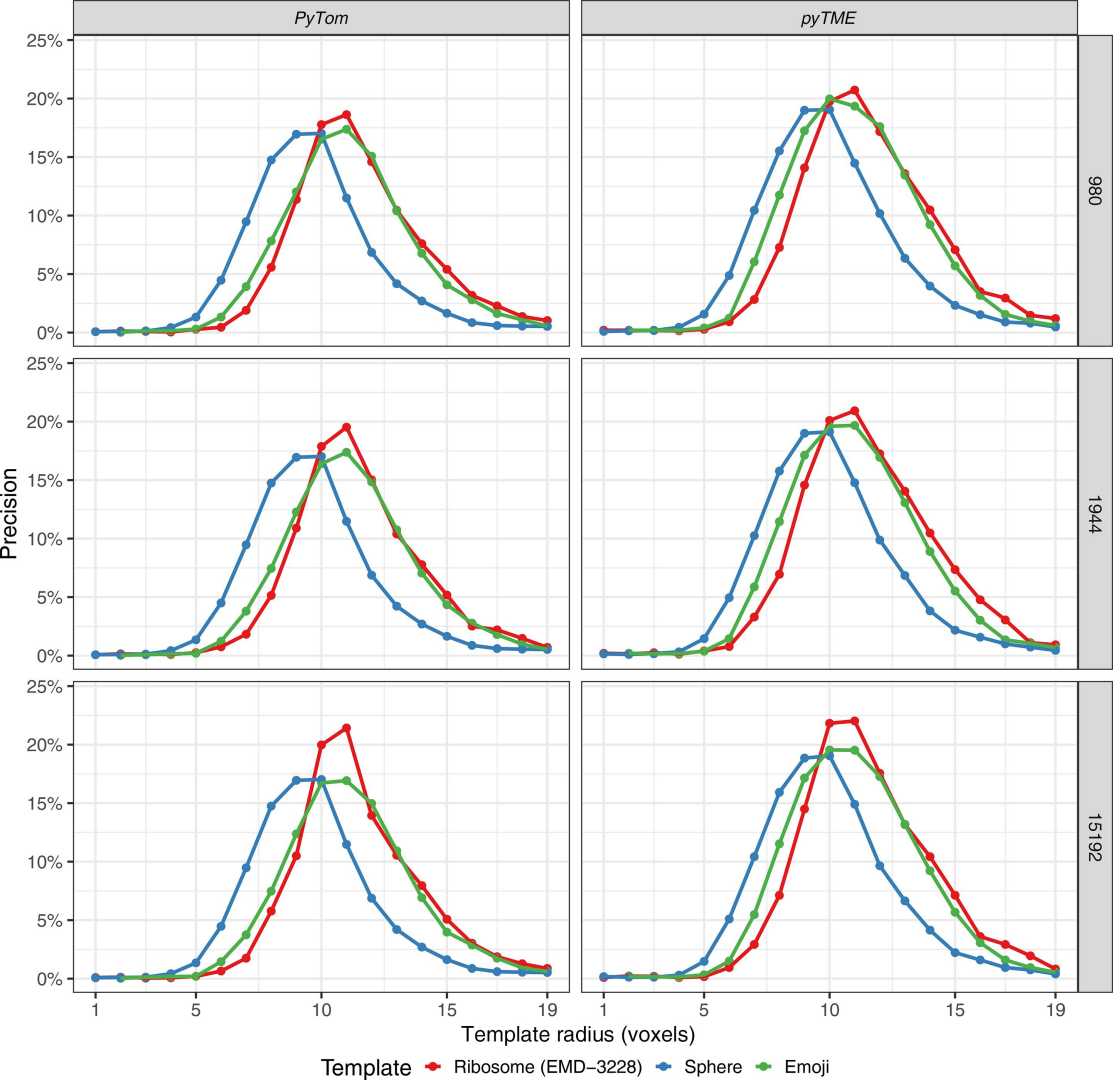
Proportion of true positives out of all picked particles (precision) by radius of templates. Template-matching results obtained with *PyTom* are shown in the left column and those obtained with *pyTME* are on the right. In each row, the sampled number of angles is shown: namely, 980, 1944 and 15 192. 4000 picks were considered when determining the precision.

When comparing the different template radii, we observed that the radius, not the chosen template, had the largest effect on the overall precision (Figs. 2 ▸ *b* and 3 ▸). The template-matching precision at 4000 picks was maximal at around 10–11 voxels, which is in line with the size of ribosomes contained in the tomogram. Templates smaller than a radius of 10 voxels performed considerably worse. This is likely due to the presence of noise or additional macromolecules that are smaller than the ribosomes but are composed of comparable density. This is in line with the fact that many studies perform template matching with large macromolecules including, but not limited to, ribosomes (de Teresa-Trueba *et al.*, 2023 ▸; Pfeffer *et al.*, 2012 ▸; Hrabe *et al.*, 2012 ▸; Chaillet *et al.*, 2023 ▸; Cruz-León *et al.*, 2023 ▸), proteasomes (Frangakis *et al.*, 2002 ▸; Nickell *et al.*, 2007 ▸), thermosomes (Frangakis *et al.*, 2002 ▸) and RuBisCO (Engel *et al.*, 2015 ▸). We also note that these results were independent of the software used, as *PyTom* and *pyTME* resulted in near-identical precision (Fig. 3 ▸).

### 3.2. Angular sampling does not improve precision

We also tested the effect of varying angular sampling on the result to ensure that undersampling did not affect the results (Fig. 3 ▸). A higher angular sampling with 15 192 angles, compared with the 1944 angles used above, did not significantly change the differentiation between the shapes and only increased the precision by approximately 3%. This indicates that while for purified, *in vitro* samples (Chaillet *et al.*, 2023 ▸) higher angular sampling improves the results at a 13 Å voxel size, *in situ* samples do not benefit from higher angular sampling to the same extent. In this case, an increase in precision by 5% does not warrant the use of approximately 15 times more computational resources. This is also to be expected since the cross-correlation score does not scale exponentially, and subtle increases in the score do not necessarily increase the differentiation from similar-sized and similar-shaped objects in the *in situ* sample.

We also cross-validated these results with *pyTME* (Maurer *et al.*, 2024 ▸) to ensure that software-specific implementation details did not affect this result. The results from both packages were near-identical across all sampled conditions.

Based on these findings, we suggest initially filtering candidate positions with low angular sampling, potentially even using a simple shape-based template of appropriate size, and sampling the same positions at a lower binning or removing false-positive hits through classification with other tools such as *RELION* (Kimanius *et al.*, 2016 ▸). Such workflows have previously been proposed in packages such as *nextPyP* (Liu *et al.*, 2023 ▸), *TomoBEAR* (Balyschew *et al.*, 2023 ▸) and *Dynamo* (Castaño-Díez *et al.*, 2017 ▸). Specifically, in this case using a spherical template is computationally most efficient because it is rotationally invariant and thus yields the best time to solution as it can be run without any angular sampling.

### 3.3. Ribosome and fatty-acid synthase are not discernible with conventional template matching

**Figure 4:**
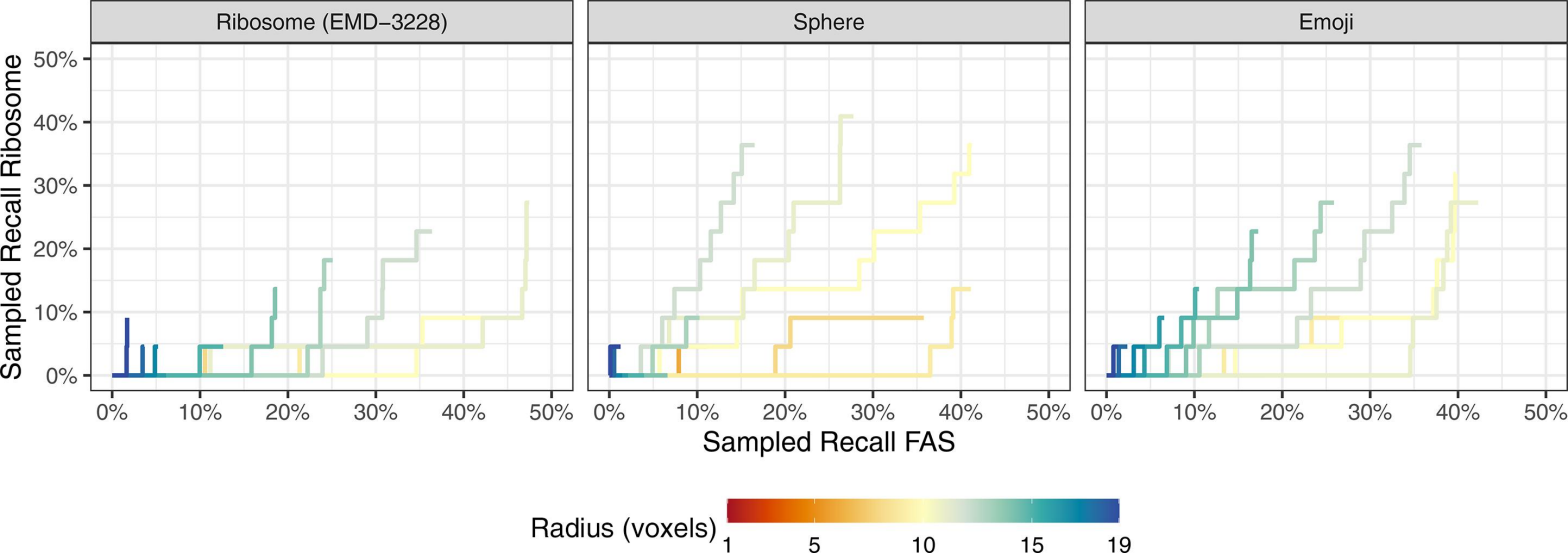
Template-matching performance on the FAS complex using three distinct templates. Picked particles were one-to-one assigned to the union of ground-truth FAS and ribosome coordinates using linear sum assignment. Each particle is assigned to no more than one class and is considered to correctly pick that class if it is within a five-voxel distance of its assigned ground-truth particle.

Finally, we assessed whether by using the ribosome, sphere and emoji templates at different radii, we could pick the FAS protein complex, which has a shape that differs substantially from that of the ribosome but has a similar size. Although the number of annotated FAS in the particular tomogram is only 22, FAS particles are among the 4000 highest scoring picks across templates and radii (Fig. 4 ▸). Although the low number of annotated FAS impedes quantitative claims, the trends are clear. For a sphere, as many as 40% of FAS are recovered, and even with the ribosome as a template more than 45% of annotated FAS instances are recovered. This finding further highlights that molecular details play a minor role in template matching at our voxel size of 13.48 Å. In our case FAS and ribosomes are similarly sized, resulting in fairly similar scores and thus poor differentiation between the two. Generally, this indicates that low-abundance proteins cannot practically be identified with sufficient precision if many other macromolecules of similar size are present.

### 3.4. Theory

We now aim to rationalize our empirical observations by examining the analytical form of the Fourier transforms of several geometric shapes and discussing them in the context of cross-correlation calculation. Based on our assessment, we conclude that template matching on the typically used 4–8 times binning is primarily driven by shape and size and list the associated implications.

Most template-matching programs, including *PyTom* (Hrabe *et al.*, 2012 ▸) and *pyTME* (Maurer *et al.*, 2024 ▸), use the cross-correlation theorem to determine the similarity between a target *f* and a template *g* at a given translation *n*, 

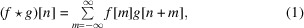

where 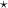 is the correlation operator. Cross-correlation is the sum of the element-wise product of the template and the target, subject to implementation-specific normalization procedures. In practice, this procedure is repeated for a set of rotations of the template.

The computational complexity of the cross-correlation operation on two identical cubes with edge length *N* is 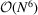 , but in practice template-matching tools reduce the complexity to 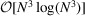 . This is achieved by expressing the cross-correlation in the spatial domain as multiplication in the Fourier domain through the cross-correlation theorem, 

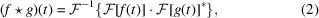

where 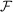 and 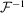 denote the forward and inverse Fourier transform and * denotes the complex conjugate. To build some intuition on how this impacts template matching, let us consider the case *g*(*t*) = *f*(*t* − *n*), where *g* differs from *f* only by a translation *n*,

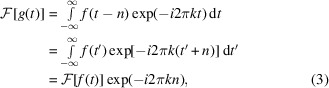

where *k* is the wavenumber in the Fourier domain and *t* is the position vector in the real domain. From this, it becomes apparent that a shift in the spatial domain corresponds to a frequency-dependent phase shift in the Fourier domain. Since |exp(−*i*2π*kn*)| = 1, the magnitude of the Fourier transform is independent of the phase shift. The cross-correlation in the real domain can be obtained by inverse Fourier transform of the element-wise product of amplitudes *A* and the sum of phases ψ,

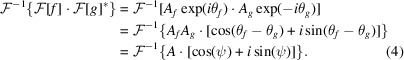

The maximum attainable cross-correlation score depends on *A*, while ψ contains the mapping between translation and realized score. As per equation (3), ψ = −2π*kn*, which results in a score *A* at translation *n*.

Above, we considered the ideal case in which the template is a shifted version of the target. In practice, this rarely holds and the template rather approximates the amplitude and phase spectrum of the target sufficiently well. Therefore, previous research has seen the use of geometric shapes for template matching, such as spheres for localization of ribosomes or RuBisCO (Engel *et al.*, 2015 ▸), cylinders for nucleosomes (Cai *et al.*, 2018 ▸) or proteasomes (Nickell *et al.*, 2007 ▸), and rectangles for membranes (Lebbink *et al.*, 2007 ▸). Intuitively, geometric shapes can be used for template matching if they approximate the structure of interest sufficiently well in the given data. However, why this is the case has not been shown explicitly. We aim to do so in the following and start by deriving the Fourier transforms of the aforementioned geometric shapes.

A sphere of radius *R* centered around the origin can be defined in real space as 

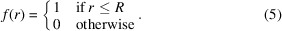

Here, *r* represents the magnitude of the position vector, *i.e.* the Euclidean distance from the origin. All points with a distance less than or equal to *R* are occupied by the sphere. Since the sphere is a real symmetric function, its Fourier transform is also real and follows as (Friedman, 1997 ▸)

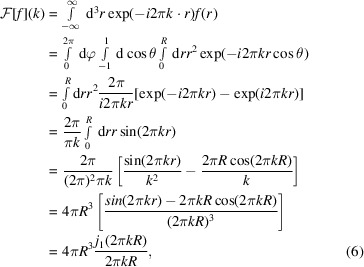

where *j*_1_(*x*) is the spherical Bessel function of first kind and order defined as

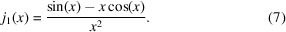




A one-dimensional rectangle, *i.e.* a box function, can be defined in real space as

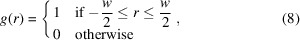

where *w* is the width of the box function. The Fourier transform of the one-dimensional box function *g*(*r*) is

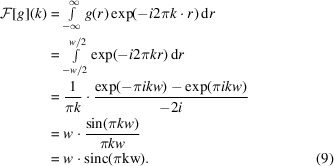

The definition of the box-function Fourier transform can be used to synthesize the Fourier transform of three-dimensional rectangles with width *a*, *b* and *c* as

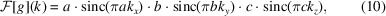

where *k*_*x*_, *k*_*y*_ and *k*_*z*_ are the wavenumbers corresponding to the spatial dimensions *x*, *y* and *z*, respectively.

The cylinder is essentially a combination of a circle and a box function and can be defined as

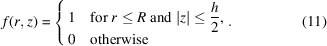

where *R* is the radius of the circle and *h* is the width of the box function. We can make use of the cylindrical symmetry and the separability of the Fourier transform to derive the closed form of the cylinder Fourier transform as follows:

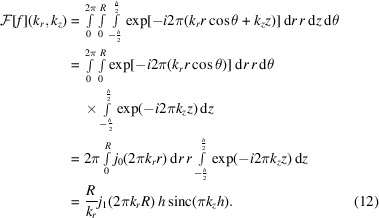

The Fourier transforms of the sphere, rectangle or cylinder either contain a Bessel function, a sinc function or a combination thereof. Therefore, these geometric shapes concentrate most of their Fourier energy in low-frequency components and dampen with a shape-specific rate towards higher frequencies. Böhm *et al.* (2000 ▸) have already hinted at the fact that low frequencies are essential for particle identification and have discussed the detection limits related to high binning.

To use geometric shapes in template matching, the macromolecule of interest within tomograms would also need to concentrate the majority of Fourier space energy in low-frequency components in a similar manner to yield a high cross-correlation score. Since low-frequency components generally recapitulate the shape and size of the analyzed object in real space, macromolecules have been template-matched by geometric shapes with similar sizes (Engel *et al.*, 2015 ▸; Cai *et al.*, 2018 ▸; Lebbink *et al.*, 2007 ▸; Nickell *et al.*, 2007 ▸). The voxel size of the tomogram used in this study was 13.48 Å, following common practices in the field (de Teresa-Trueba *et al.*, 2023 ▸; Xue *et al.*, 2022 ▸; Engel *et al.*, 2015 ▸; Cai *et al.*, 2018 ▸; Frangakis *et al.*, 2002 ▸; Chaillet *et al.*, 2023 ▸; Wan *et al.*, 2024 ▸; Rice *et al.*, 2023 ▸; Genthe *et al.*, 2023 ▸; Hoffmann *et al.*, 2022 ▸). Therefore, no features smaller than ∼27 Å can be represented without artifacts according to the Shannon–Nyquist sampling theorem (Shannon, 1949 ▸). 27 Å is in excess of most detailed structural features in a macromolecule. Consequently, the majority of Fourier space energy is also concentrated in low-frequency components, analogous to the discussed geometric shapes.

**Figure 5:**
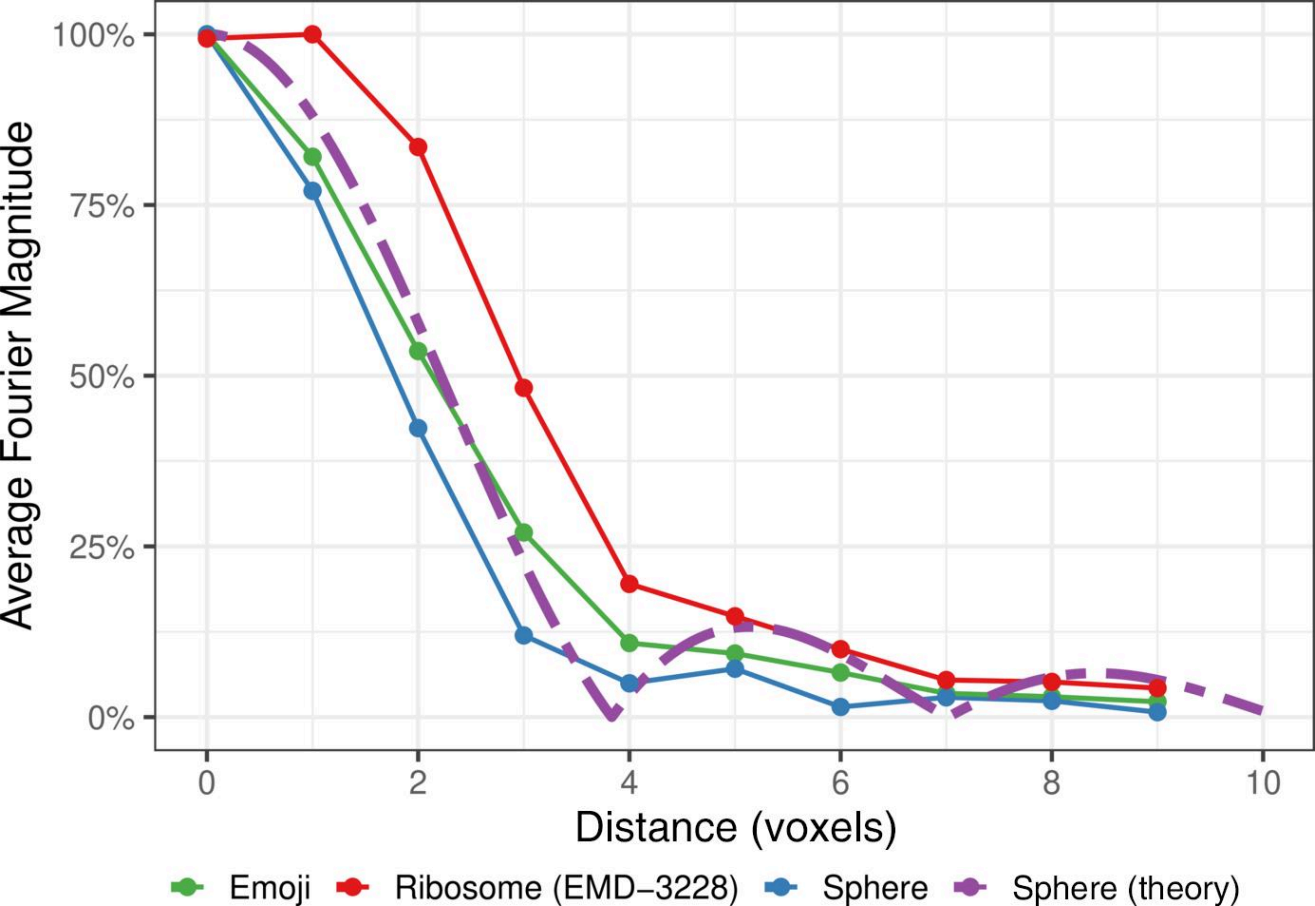
Fourier magnitude spectrum averages by ‘distance’ and template. ‘Distance’ was computed as the Euclidean distance from the zero-frequency component and was rounded to the nearest integer. Templates used for template matching at a radius of 10 voxels are shown (Fig. 1 ▸). ‘Sphere (theory)’ refers to the theoretical derivations made in Section [Sec sec3.4]3.4 with *R* = 10 (Friedman, 1997 ▸). Magnitude spectrum averages were linearly scaled to the interval [0, 1] to facilitate curve-shape comparison.

We computed the radially averaged Fourier magnitude spectrum for three templates at a radius of 10 voxels and compared them with the theoretical curve of a sphere (Fig. 5 ▸). The average magnitude of a template *g* at a Euclidean distance *d* from the zero-frequency component of the Fourier transform was computed as 

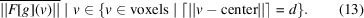

We observed that the templates used here are primarily composed of the same low-frequency components. This matches the theoretical assumption that template matching using geometric shapes is possible if the majority of the Fourier space energy is concentrated similarly. Accordingly, we see little differentiation in the total precision achieved for varying templates with the same radius and most variation between the same template with varying radii. While the templates assessed here approximate the phase spectrum of the ribosome sufficiently well to avoid cancelation and serve as a template, the phase difference must be considered in practice (see equation 4).

These theoretical considerations have three important implications for template matching. (i) Since template matching at this binning is primarily about matching object size, macromolecules of similar size to the macromolecule of interest will be identified as false positives. (ii) Small macromolecules would mainly be represented through high frequencies, which overlap with noise in the data. This relation makes template-matching small macromolecules at this binning near-impossible. (iii) More accurate templates are unlikely to improve the template-matching performance because high-resolution information cannot be accurately represented at the typically used 4–8 times binning.

## 4. Conclusions and outlook

In this article, we explored the effect of shape, size and angular sampling on the precision of matching ribosomes in an annotated *S. cerevisiae* tomogram at the commonly used four-times binning (de Teresa-Trueba *et al.*, 2023 ▸; Xue *et al.*, 2022 ▸; Engel *et al.*, 2015 ▸; Cai *et al.*, 2018 ▸; Frangakis *et al.*, 2002 ▸; Chaillet *et al.*, 2023 ▸; Wan *et al.*, 2024 ▸; Rice *et al.*, 2023 ▸; Genthe *et al.*, 2023 ▸; Hoffmann *et al.*, 2022 ▸). We showed that using a ribosome subtomogram average, a sphere and a heart emoji as a template resulted in near-identical performance in our benchmark data set, highlighting the shortcomings and limitations of using highly binned tomograms for template matching. We show, based on theoretical arguments, that because highly binned tomograms primarily consist of low-frequency information, geometric shapes such as spheres, cylinders or rectangles of appropriate size can be used to identify macromolecules equally well as detailed structural templates. Therefore, cross-correlation scores are primarily driven by the shape and size of the template, rather than its internal structure, as seen in our practical experiments. This has important implications when moving to more complex data sets or smaller target structures in the future. At high binning, macromolecules of similar size will often be identified as false positives over the macromolecule of interest, regardless of how detailed the template is. Importantly, this issue will be more pronounced for small molecules, where the high frequencies will overlap with noise, and template quality will not improve the performance either.

Based on these considerations, we suggest the following moving forward. Firstly, cross-correlation-based scoring methods appear to be a suboptimal measure of similarity in tomograms. This is particularly apparent for high binnings. Therefore, different, perhaps nonlinear, similarity metrics such as those used in machine learning can enhance template-matching performance (Moebel *et al.*, 2021 ▸; de Teresa-Trueba *et al.*, 2023 ▸; Rice *et al.*, 2023 ▸; Genthe *et al.*, 2023 ▸). However, for small macromolecules, generating adequate training data sets could be highly challenging, as manual curation would be limited by noise levels and the visibility of macromolecules by eye. Secondly, analyzing lower binned tomograms can potentially improve cross-correlation-based template matching. The first developments have recently emerged in both 2D and 3D. 2D template matching (Rickgauer *et al.*, 2017 ▸; Lucas *et al.*, 2021 ▸) avoids the high computational cost associated with exhaustive sampling, and high-resolution matching at low binning in 3D has recently been reported and demonstrated to give fewer false-positive results (Cruz-León *et al.*, 2024 ▸). It also becomes evident that at such high binning it is computationally most efficient to first use shape-based filtering, ideally with a spherical mask to filter candidate positions broadly (Liu *et al.*, 2023 ▸), and then further refine them locally with high-resolution template matching or by filtering false positives by classification in programs such as *RELION* (Kimanius *et al.*, 2016 ▸). To make shape-based picking easily accessible, we provide a Napari plugin via our software package *pyTME* (Maurer *et al.*, 2024 ▸) which enables the generation of spherical, cylindrical or ellipsoid templates and masks.

The high computational cost associated with 3D template matching at low binning will be overcome in the future by further developing template-matching software for efficient use on GPUs without needing to bin the reconstructed tomograms (Maurer *et al.*, 2024 ▸; Chaillet *et al.*, 2023 ▸). Similarly, higher angular sampling at lower binning might also be beneficial in specific cases (Chaillet *et al.*, 2023 ▸; Cruz-León *et al.*, 2024 ▸). Future developments will also need to tackle additional challenges such as noise and specimen motion resulting from problems with tilt alignment, sample deformation and errors in CTF correction (Voortman *et al.*, 2014 ▸; Lucas *et al.*, 2021 ▸).

Lastly, we suggest broadening benchmark entities beyond large and highly abundant globular structures such as the ribosome when evaluating new template-matching algorithms. In particular, providing test sets of particles that have similar low-frequency information is necessary to determine the discriminatory power of novel template-matching methods, score functions or processing approaches. Novel methods should also be validated against the simple geometric shapes considered here to ensure that they perform better and justify the higher computational cost.

## 5. Data availability

The tomogram and ground-truth picks are freely available from EMPIAR (EMPIAR-10988, TS_037). The scaled maps for the ribosome (EMDB entry EMD-3228), sphere, emoji and HA at various radii, the resulting picks, the raw data for plots and the scripts used are freely available on GitHub at https://github.com/maurerv/ribosomeTemplateMatching. 

## Supplementary Material

Supplementary Figure S1. DOI: 10.1107/S2059798324004303/vo5016sup1.pdf

## References

[bb1] Balyschew, N., Yushkevich, A., Mikirtumov, V., Sanchez, R. M., Sprink, T. & Kudryashev, M. (2023). *Nat. Commun.* **14**, 6543. 10.1038/s41467-023-42085-w PMC10582028 37848413

[bb2] Beck, M., Malmström, J. A., Lange, V., Schmidt, A., Deutsch, E. W. & Aebersold, R. (2009). *Nat. Methods*, **6**, 817–823. 10.1038/nmeth.1390 PMC2862215 19838170

[bb3] Bharat, T. A. M. & Scheres, S. H. W. (2016). *Nat. Protoc.* **11**, 2054–2065. 10.1038/nprot.2016.124 PMC5215819 27685097

[bb4] Böhm, J., Frangakis, A. S., Hegerl, R., Nickell, S., Typke, D. & Baumeister, W. (2000). *Proc. Natl Acad. Sci. USA*, **97**, 14245–14250. 10.1073/pnas.230282097 PMC18903 11087814

[bb5] Cai, S., Chen, C., Tan, Z. Y., Huang, Y., Shi, J. & Gan, L. (2018). *Proc. Natl Acad. Sci. USA*, **115**, 10977–10982. 10.1073/pnas.1720476115 PMC6205422 30297429

[bb7] Castaño-Díez, D., Kudryashev, M., Arheit, M. & Stahlberg, H. (2012). *J. Struct. Biol.* **178**, 139–151. 10.1016/j.jsb.2011.12.017 22245546

[bb6] Castaño-Díez, D., Kudryashev, M. & Stahlberg, H. (2017). *J. Struct. Biol.* **197**, 135–144. 10.1016/j.jsb.2016.06.005 27288866

[bb8] Chaillet, M. L., van der Schot, G., Gubins, I., Roet, S., Veltkamp, R. C. & Förster, F. (2023). *Int. J. Mol. Sci.* **24**, 13375. 10.3390/ijms241713375 PMC10487639 37686180

[bb9] Cruz-León, S., Majtner, T., Hoffmann, P. C., Kreysing, J. P., Kehl, S., Tuijtel, M. W., Schaefer, S. L., Geissler, K., Beck, M., Turoňová, B. & Hummer, G. (2024). *Nat. Commun.* **15**, 3992. 10.1038/s41467-024-47839-8 PMC11088655 38734767

[bb10] Engel, B. D., Schaffer, M., Kuhn Cuellar, L., Villa, E., Plitzko, J. & Baumeister, W. (2015). *eLife*, **4**, e04889. 10.7554/eLife.04889 PMC4292175 25584625

[bb11] Frangakis, A. S., Böhm, J., Förster, F., Nickell, S., Nicastro, D., Typke, D., Hegerl, R. & Baumeister, W. (2002). *Proc. Natl Acad. Sci. USA*, **99**, 14153–14158. 10.1073/pnas.172520299 PMC137853 12391313

[bb12] Friedman, J. (1997). *Protein Eng. Des. Sel.* **10**, 851–863. 10.1093/protein/10.8.851 9415436

[bb13] Genthe, E., Miletic, S., Tekkali, I., Hennell James, R., Marlovits, T. C. & Heuser, P. (2023). *J. Struct. Biol.* **215**, 107990. 10.1016/j.jsb.2023.107990 37364763

[bb14] Hoffmann, P. C., Kreysing, J. P., Khusainov, I., Tuijtel, M. W., Welsch, S. & Beck, M. (2022). *Nat. Commun.* **13**, 7435. 10.1038/s41467-022-34997-w PMC9718845 36460643

[bb15] Hrabe, T., Chen, Y., Pfeffer, S., Kuhn Cuellar, L., Mangold, A.-V. & Förster, F. (2012). *J. Struct. Biol.* **178**, 177–188. 10.1016/j.jsb.2011.12.003 22193517

[bb16] Iudin, A., Korir, P. K., Somasundharam, S., Weyand, S., Cattavitello, C., Fonseca, N., Salih, O., Kleywegt, G. J. & Patwardhan, A. (2022). *Nucleic Acids Res.* **51**, D1503–D1511. 10.1093/nar/gkac1062 PMC9825465 36440762

[bb17] Jumper, J., Evans, R., Pritzel, A., Green, T., Figurnov, M., Ronneberger, O., Tunyasuvunakool, K., Bates, R., Žídek, A., Potapenko, A., Bridgland, A., Meyer, C., Kohl, S. A. A., Ballard, A. J., Cowie, A., Romera-Paredes, B., Nikolov, S., Jain, R., Adler, J., Back, T., Petersen, S., Reiman, D., Clancy, E., Zielinski, M., Steinegger, M., Pacholska, M., Berghammer, T., Bodenstein, S., Silver, D., Vinyals, O., Senior, A. W., Kavukcuoglu, K., Kohli, P. & Hassabis, D. (2021). *Nature*, **596**, 583–589. 10.1038/s41586-021-03819-2 PMC8371605 34265844

[bb18] Kimanius, D., Forsberg, B., Scheres, S. H. W. & Lindahl, E. (2016). *eLife*, **5**, e18722. 10.7554/eLife.18722 PMC5310839 27845625

[bb19] Kühner, S., van Noort, V., Betts, M. J., Leo-Macias, A., Batisse, C., Rode, M., Yamada, T., Maier, T., Bader, S., Beltran-Alvarez, P., Castaño-Diez, D., Chen, W.-H., Devos, D., Güell, M., Norambuena, T., Racke, I., Rybin, V., Schmidt, A., Yus, E., Aebersold, R., Herrmann, R., Böttcher, B., Frangakis, A. S., Russell, R. B., Serrano, L., Bork, P. & Gavin, A.-C. (2009). *Science*, **326**, 1235–1240. 10.1126/science.1176343 19965468

[bb20] Lebbink, M., Geerts, W. J. C., van der Krift, T. P., Bouwhuis, M., Hertzberger, L. O., Verkleij, A. J. & Koster, A. J. (2007). *J. Struct. Biol.* **158**, 327–335. 10.1016/j.jsb.2006.12.001 17270464

[bb21] Liu, H.-F., Zhou, Y., Huang, Q., Piland, J., Jin, W., Mandel, J., Du, X., Martin, J. & Bartesaghi, A. (2023). *Nat. Methods*, **20**, 1909–1919. 10.1038/s41592-023-02045-0 PMC10703682 37884796

[bb22] Lucas, B. A., Himes, B. A., Xue, L., Grant, T., Mahamid, J. & Grigorieff, N. (2021). *eLife*, **10**, e68946. 10.7554/eLife.68946 PMC8219381 34114559

[bb23] Lučić, V., Rigort, A. & Baumeister, W. (2013). *J. Cell Biol.* **202**, 407–419. 10.1083/jcb.201304193 PMC3734081 23918936

[bb24] Mahamid, J., Pfeffer, S., Schaffer, M., Villa, E., Danev, R., Kuhn Cuellar, L., Förster, F., Hyman, A. A., Plitzko, J. & Baumeister, W. (2016). *Science*, **351**, 969–972. 10.1126/science.aad8857 26917770

[bb25] Maurer, V. J., Siggel, M. & Kosinski, J. (2024). *SoftwareX*, **25**, 101636.

[bb26] Moebel, E., Martinez-Sanchez, A., Lamm, L., Righetto, R. D., Wietrzynski, W., Albert, S., Larivière, D., Fourmentin, E., Pfeffer, S., Ortiz, J., Baumeister, W., Peng, T., Engel, B. D. & Kervrann, C. (2021). *Nat. Methods*, **18**, 1386–1394. 10.1038/s41592-021-01275-4 34675434

[bb27] Nickell, S., Mihalache, O., Beck, F., Hegerl, R., Korinek, A. & Baumeister, W. (2007). *Biochem. Biophys. Res. Commun.* **353**, 115–120. 10.1016/j.bbrc.2006.11.141 17173858

[bb28] Pfeffer, S., Brandt, F., Hrabe, T., Lang, S., Eibauer, M., Zimmermann, R. & Förster, F. (2012). *Structure*, **20**, 1508–1518. 10.1016/j.str.2012.06.010 22819217

[bb29] Pfeffer, S., Dudek, J., Schaffer, M., Ng, B. G., Albert, S., Plitzko, J., Baumeister, W., Zimmermann, R., Freeze, H. H., Engel, B. D. & Förster, F. (2017). *Nat. Commun.* **8**, 14516. 10.1038/ncomms14516 PMC5321747 28218252

[bb30] Pyle, E. & Zanetti, G. (2021). *Biochem. J.* **478**, 1827–1845. 10.1042/BCJ20200715 PMC8133831 34003255

[bb31] Rice, G., Wagner, T., Stabrin, M., Sitsel, O., Prumbaum, D. & Raunser, S. (2023). *Nat. Methods*, **20**, 871–880. 10.1038/s41592-023-01878-z PMC10250198 37188953

[bb32] Rickgauer, J. P., Grigorieff, N. & Denk, W. (2017). *eLife*, **6**, e25648. 10.7554/eLife.25648 PMC5453696 28467302

[bb33] Shannon, C. E. (1949). *Proc. IRE*, **37**, 10–21.

[bb34] Tang, G., Peng, L., Baldwin, P. R., Mann, D. S., Jiang, W., Rees, I. & Ludtke, S. J. (2007). *J. Struct. Biol.* **157**, 38–46. 10.1016/j.jsb.2006.05.009 16859925

[bb35] Teresa-Trueba, I. de, Goetz, S. K., Mattausch, A., Stojanovska, F., Zimmerli, C. E., Toro-Nahuelpan, M., Cheng, D. W. C., Tollervey, F., Pape, C., Beck, M., Diz-Muñoz, A., Kreshuk, A., Mahamid, J. & Zaugg, J. B. (2023). *Nat. Methods*, **20**, 284–294. 10.1038/s41592-022-01746-2 PMC9911354 36690741

[bb36] Volkmann, N. (2010). *Methods Enzymol.* **483**, 31–46. 10.1016/S0076-6879(10)83002-2 20888468

[bb37] Voortman, L. M., Vulović, M., Maletta, M., Voigt, A., Franken, E. M., Simonetti, A., Peters, P. J., van Vliet, L. J. & Rieger, B. (2014). *J. Struct. Biol.* **187**, 103–111. 10.1016/j.jsb.2014.06.007 24998892

[bb38] Wan, W., Khavnekar, S. & Wagner, J. (2024). *Acta Cryst.* D**80**, 336–349. 10.1107/S205979832400295X PMC11066880 38606666

[bb39] Wan, W., Khavnekar, S., Wagner, J., Erdmann, P. & Baumeister, W. (2020). *Microsc. Microanal.* **26**, 2516.

[bb40] Wilfling, F., Lee, C.-W., Erdmann, P. S., Zheng, Y., Sherpa, D., Jentsch, S., Pfander, B., Schulman, B. A. & Baumeister, W. (2020). *Mol. Cell*, **80**, 764–778. 10.1016/j.molcel.2020.10.030 PMC7721475 33207182

[bb41] Wu, X., Zeng, X., Zhu, Z., Gao, X. & Xu, M. (2019). *Computational Biology*, edited by H. Husi, pp. 175–186. Brisbane: Codon Publications. 31815381

[bb42] Xue, L., Lenz, S., Zimmermann-Kogadeeva, M., Tegunov, D., Cramer, P., Bork, P., Rappsilber, J. & Mahamid, J. (2022). *Nature*, **610**, 205–211. 10.1038/s41586-022-05255-2 PMC9534751 36171285

[bb43] Zhang, K., Lucas, B. & Grigorieff, N. (2023). *Microsc. Microanal.* **29**, 931.

