# Supplementary material for: What shapes template-matching performance in cryogenic electron tomography in situ?
Source: Acta Crystallogr D Struct Biol. 2024 May 28;80(Pt 6):410–20. doi: 10.1107/S2059798324004303 (PMC11154592; doi:10.1107/S2059798324004303)
Supplement: Supplementary file 1 [file d-80-00410-sup1.pdf]

STRUCTURAL BIOLOGY

Volume 80 (2024)

Supporting information for article:

What shapes template-matching performance in cryogenic electron tomography *in situ*?

Valentin J. Maurer, Marc Siggel and Jan Kosinski

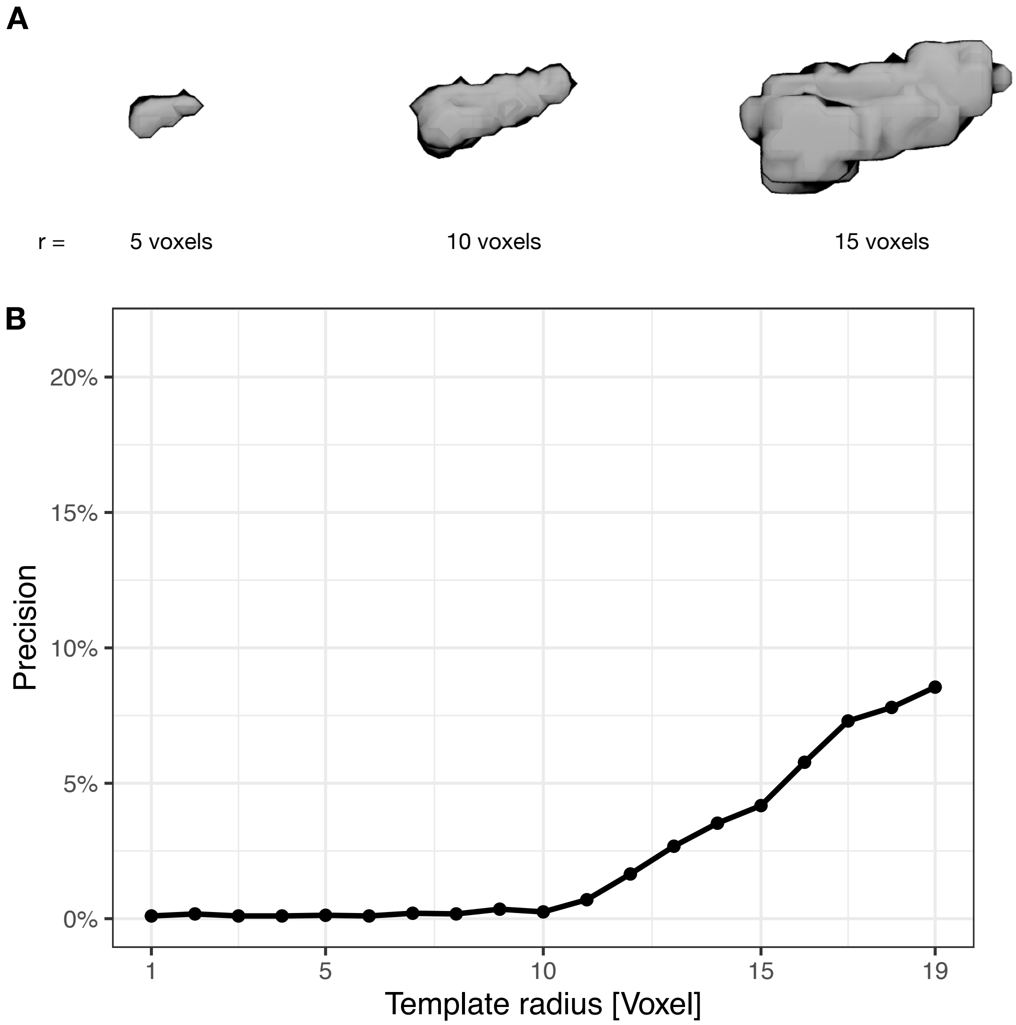

Fig. S1. **A** Snapshots of the hemagglutinin (HA) templates with different radii used as a control. **B** Proportion of true positives out of all picked particles (precision) by the radius of templates. The HA structure was matched with 1.944 angles.
